# Mechanism of N-acetylcysteine in alleviating diabetic myocardial ischemia reperfusion injury by regulating PTEN/Akt pathway through promoting DJ-1

**DOI:** 10.1042/BSR20192118

**Published:** 2020-06-04

**Authors:** Wenyuan Li, Wei Li, Yan Leng, Yonghong Xiong, Rui Xue, Rong Chen, Zhongyuan Xia

**Affiliations:** 1 Department of Anesthesiology, Renmin Hospital of Wuhan University, Wuhan 430060, China; 2 Department of Anesthesiology, Renmin Hospital, Hubei University of Medicine, 442000, Hubei, China

**Keywords:** diabetes, DJ-1, myocardial ischemia reperfusion injury, N-acetyl-L-cysteine, PTEN/Akt pathway

## Abstract

Ischemic heart disease is the main cardiovascular complication of diabetes patients which is mainly caused by oxidative stress. DJ-1 is the key regulator for myocardial protection through inhibiting phosphatase and tensin homolog deleted on chromosome 10 (PTEN) and activating Akt (also known as PKB or protein kinase B). This research is to investigate whether the antioxidant N-acetylcysteine (NAC) could alleviate diabetic myocardial ischemia/reperfusion (I/R) injury by the protective molecule DJ-1. DJ-1 in rat myocardial H9c2 cells and cardiac tissue was respectively knocked down by siRNA and adeno-associated virus (AAV). From the present study, it could be found that compared with high glucose (HG)-normal (N)/DM group, hypoxia/reoxygenation (H/R) or I/R injury can aggravate oxidative stress injury and apoptosis rate of myocardial cells, inhibit the expression of Bcl-2, activate the BAX and cleaved caspase-3 (c-caspase-3) protein and PTEN/Akt pathway. However, in the groups of HG-N, DM, HG-N+I/R and DM+I/R, NAC can significantly reduce oxidative stress injury and apoptosis rate of myocytes, promote the Bcl-2 and DJ-1 molecules, inhibit BAX and c-caspase-3 protein and PTEN/Akt pathway. Compared with HG-N+I/R+NAC and DM+I/R+NAC groups, the oxidative stress injury, apoptosis rate of myocardial cells and heart tissues increased after the knockdown of DJ-1, the expression of Bcl-2 and DJ-1 were inhibited, the BAX and c-caspase-3 expression was increased, and PTEN/Akt pathway was activated. Taken together, the findings suggest that NAC can reduce I/R injury in diabetic myocardium by up-regulating the PTEN/Akt pathway through the level of DJ-1.

## Introduction

Diabetes is an independent risk factor for cardiovascular disease. The vulnerability of myocardial ischemia/reperfusion (I/R) injury in diabetic patients is increased [1,2]. The International Diabetes Federation (IDF) estimates that the total prevalence of diabetes in 2011 is 366 million and is expected to increase to 552 million by 2030 [3]. Evidence-based medicine shows that 75% of death patients are closely related to ischemic coronary artery disease. Therefore, exploring the molecular mechanism of diabetic myocardial I/R injury and formulating effective prevention and treatment measures can fundamentally reduce the perioperative complications of diabetes patients, reduce mortality, and improve postoperative cardiac function and quality of life.

The *DJ-1* gene is an oncogene firstly discovered in NIH3T3 cells in 1997, and its encoded protein is widely expressed in various tissues [4]. It participates in a variety of physiological and pathological activities such as antioxidant [5], molecular chaperone [6], inhibition of apoptosis [7], regulation of androgen receptors [8]. Mitochondria are important sites for oxidative stress, and DJ-1 protein is closely related to mitochondria. Although DJ-1 protein is less distributed in mitochondria, DJ-1 protein located in mitochondria has a stronger cellular protective effect than DJ-1 protein located in cytoplasm and nucleus [9]. Mitochondrial dysfunction was found in DJ-1 gene knockout mice, mainly including decreased activity of mitochondrial complex I and decreased mitochondrial membrane potential [10]. Under the stimulation of oxidative stress, DJ-1 protein can decrease the protein expression of BAX by reducing the transcriptional activity of p53, and then inhibit the apoptosis pathway of BAX-caspases, so as to protect mitochondrial function [11]. When genetic mutation occurred or DJ-1 protein level decreased, cellular antioxidant capacity is reduced, thus, the sensitivity of cells to oxidative stress was increased, the homeostasis of intracellular REDOX was out of balance and ROS accumulation in great quantities, which in turn leads to oxidative stress and damage mitochondria steady-state, ATP synthesis reduced, the further increase in mitochondria and cell protein, lipid and DNA damage [12]. As an important negative regulator of phosphatase and tensin homolog deleted on chromosome 10 (PTEN), DJ-1 promotes the activation of phosphoinositide 3-kinase (PI-3K)/Akt (also known as PKB or protein kinase B) and produces myocardial protection [13].

N-acetylcysteine (NAC) is a thiol-containing free radical scavenger and precursor to the antioxidant glutathione (GSH), and is therefore widely used to remove ROS from oxidative stress [14]. Available evidence suggests that NAC has a protective effect on myocardial I/R injury [15]. At the same time, our previous study found that NAC can also reduce myocardial I/R injury in diabetic by caveolin-3/endothelial nitric oxide synthases (eNOSs) signaling pathway, but not explain whether NAC can attenuate myocardial damage during I/R in diabetic by regulating DJ-1 expression [14,16]. Therefore, this experiment first examined whether DJ-1 may be involved in the pathophysiological process of diabetic myocardial I/R injury through the PTEN/Akt pathway. Again, it was tested whether NAC can attenuate diabetic myocardial I/R injury by modulating DJ-1/PTEN/Akt signaling.

## Materials and methods

### Reagents

Normal myocardial H9c2 cell line was purchased from China Center for Type Culture Collection (Wuhan University). Dulbecco’s modified Eagle’s medium (DMEM) low-glucose medium (sugar concentration 5.5 mmol/l) and 100 μ/ml penicillin + 0.1 g/l streptomycin double antibiotic were purchased from Gibco (Grand Island, NY). Fetal bovine serum was purchased from Sijiqing (China). Trypsin was purchased from GSEE-TECH (China). DJ-1, cleaved caspase-3 (c-caspase-3,) PTEN, Akt, p-Akt, Bcl-2, BAX and GAPDH primary antibodies were purchased from CST (U.S.A.). The Prime-Script RT reagent kit, SYBR Premix Ex Taq kit and TRIzol were purchased from TAKARA (China). Fluorescent secondary antibody IRDye800CW and Odyssey Infrared Imaging System were purchased from LI-COR (U.S.A.). Victor X-type microplate reader was purchased from PerkinElmer (U.S.A.). Flow kit was purchased from Nanjing built (China). Flow cytometry was purchased from BD (U.S.A.).

### Cell culture and administration

The normal growth logarithmic H9c2 cardiomyocytes were randomly divided into five groups: high glucose (HG) and normoxia group (HG-N), HG hypoxia/reoxygenation group (HG-H/R), HG normoxia + NAC group (HG-N+NAC), HG hypoxia/reoxygenation + NAC group (HG-H/R + NAC), HG hypoxia/reoxygenation + NAC + siRNA-DJ-1 group (HG-H/R + NAC + siRNA-DJ -1). The cells in each group were cultured in a common incubator (volume fraction 90% atmosphere + volume fraction 10% carbon dioxide (CO_2_). The cells in HG-H/R + NAC + siRNA-DJ-1 group were given siRNA (Santa Cruz Biotechnology) knockdown cells before modeling and intervention. The DJ-1 molecule was then pretreated with siRNA in the HG-I/R+NAC group cells for 24 h before NAC modelling, and the HG model was prepared by adding HG medium for 24 h after 24 h. The HG-H/R cell model was placed in a three-gas incubator (volume fraction 94% N_2_ + volume fraction 5% CO_2 +_ volume fraction 1% O_2_) for hypoxia for 4 h, and the H/R model was prepared by reoxygenation for 2 h in a common incubator.

### Preparation and intervention of diabetic myocardial I/R rat model

Fifty SPF adult male Sprague–Dawley (SD) rats weighing 210–240 g were purchased from Beijing Huakang Biotechnology Co., Ltd. The animal experiment was conducted in the animal experiment center of Renmin Hospital of Wuhan University at temperature of 25 ± 2°C, relative humidity of 50 ± 15% and normal circadian rhythm (12-h dark/12-h light). All study protocols were in accordance with internationally accepted principles and the Guidelines for the Care and Use of Laboratory Animals of Wuhan University (Wuhan, China), approved by the Ethics Committee of Renmin Hospital of Wuhan University and consistent with the Ethical Guidelines of the International Association for Pain Research. The animal experiment number was SYXK (HUBEI) 2014-0080. Animal quality certificate number was NO.11400700265563. All the rats were randomly divided into five groups: diabetic mellitus group (DM), diabetic myocardial I/R group (DM-I/R), diabetes + NAC intervention group (DM + NAC), diabetic myocardial I/R + NAC intervention group (DM-I/R + NAC), diabetic myocardial I/R +NAC intervention +DJ-1 adeno-associated virus (AAV) knockdown group (DM-I/R + NAC + AAV-DJ-1). DM-I/R + NAC + AAV-DJ-1 rats were targeted to knock down the expression of DJ-1 in rat hearts using tail vein injection prior to intervention.

To prepare diabetic rat model, the rats were injected with 1% of STZ (60 mg/kg) once in the tail vein after fasting for 12 h, and fasted for 6 h after 3 days. The fasting blood glucose level was greater than 16.7 mmol/l prompted that the diabetes model had been successfully modelled. Thereafter, the amount of water drunk and food eaten in the rats were recorded daily, and blood glucose and body weight were monitored weekly. Establishment of a DJ-1 silent rat model using serotype AAV9 (Hanbio Biotechnology Co., Ltd.) tail vein injection. One week after successful induction of the diabetes model, rats in the DM + NAC, DM-I/R + NAC and DM-I/R + NAC + AAV-DJ-1 groups were treated with a dose of 1.5 g/kg.d NAC for 4 weeks.

To prepare myocardial I/R rat model, rats were fasted for 12 h before operation, anesthetized by intraperitoneal injection of 1% pentobarbital sodium 60 mg/kg and fixed on the homeothermic table to keep 37°C core body temperature, connected to electrocardiograph (ECG) monitoring, connected after tracheal intubation. The ventilator mechanically controls breathing. The venous access was established by separating the right internal jugular vein. The right femoral artery was inserted to record the invasive arterial pressure. The needle electrode was inserted under the skin and chest of the rat, and the small animal ECG was connected. A 75% ethanol solution was used to disinfect the chest skin, and a 1-cm incision was made in the left side of the sternum. The layers were separated and exposed, and the third and fourth ribs were cut. The hooks were opened along the intercostal space, and the capsule was cut to fully expose the heart. A ligature was placed ∼2 mm below the branch of the left anterior descending coronary artery (LAD) of the coronary artery with a 6-0 lesion-free line for ligation. At the time of ligation, a thin plastic tube having a diameter of 0.2 cm was used to pass through the ligature wire and clamped with a microscopic hemostat. After 10 min of stabilization, LAD was ligated to cause myocardial ischemia. After 30 min, the ligature was released and re-perfused for 2 h. All rats were killed by cervical dislocation after 2 h of reperfusion. The success criteria for LAD ligation were: whitening of the anterior region, ST-segment elevation in the ECG II lead (T-wave towering, fusion with QRS waves, QRS wave broadening, high). The criteria for reperfusion success are: ST segment fall, apex redness.

### SiRNA down-regulates DJ-1 molecule in H9c2 cells

H9c2 cells were seeded in six-well plates. A 1 × 10^5^ cells were added to each well, and cultured until cell density was 50%, then the cell transfection was performed. The siRNA was diluted with the optimized medium and mixed; the liposomes were diluted and diluted for 10 min at room temperature; the diluted siRNA was thoroughly mixed with the liposomes and incubated for 25 min at room temperature. One milliliter of the fully mixed liposome was added to the culture dish and placed in a 37°C incubator for 6 h. After siRNA transfection for 6 h, the medium was discarded, and the appropriate amount of the medium without the anti-antibiotic was added and placed in a 37°C cell culture incubator. Continue to culture for 24 h. The corresponding drug treatment and modeling intervention were given. The siRNA was provided by Gena Pharma, and the sequence (5′–3′) was CGAGCUGGGAUUAAAGUCATTUGACUUUAAUCCCAGCUCGTT.

### Cell counting kit-8 kit for measuring cell viability

H9c2 cardiomyocytes were collected by 0.25% trypsin digestion, and seeded in 96-well culture plates at 5000 cells per well. After incubation at 37°C in a 5% carbon dioxide incubator for 24 h, the cells in each group were given the corresponding intervention. The 96-well plate aspirated the drug-containing medium and 10 μl of cell counting kit-8 (CCK-8) solution were added per well and 90 μl of serum-free medium, and incubated it at 37°C for 4 h, then measured the absorbance at 450 nm.

### Determination of LDH, GSH, CK-MB and 15-F2t-Isoprostane content

The supernatant, lysate and serum samples of each group were collected, centrifuged at 4°C, 3000 rpm for 15 min, and the supernatant or serum was taken and stored in a refrigerator at −20°C. Specific procedures were based on the kit instructions, the levels of LDH, GSH in the cell supernatant, 15-F2t-Isoprostane (15-F2t-isop) in the cell supernatant and lysate, serum CK-MB and 15-F2t-isop were measured under a microplate reader.

### Quantitative real-time PCR to detect mRNA expression

The primers were designed and synthesized by Wuhan Qingke Biotechnology Co., Ltd (Wuhan, Hubei, China). The primer sequences for DJ-1 and GAPDH were listed as follows. DJ-1 forward and reverse primer sequences were 5′-ACCGCGCAGGAAAAACACGC-3′ and 5′-CTGCCAGACGGCTCTGCAC-3′. The housekeeping gene *GAPDH* forward and reverse primer sequences were 5′-AAC GGCACA GTCAAGGCTGA-3′ and 5′-AACGGCACAGTCAAGGCTGA-3′. Total RNA for myocardial tissue and H9c2 cells was extracted by TRIzol. According to the Prime-Script RT reagent kit and the SYBR Premix Ex Taq kit instructions, the RNA was reverse-transcribed into cDNA. Then the polymerase chain reaction initiated at 95°C for 30 s, followed 40 cycles of amplification of denaturation at 95°C for 5 s, annealing at 60°C for 34 s by using a StepOne Plus device (Applied Biosystems). The 2^−ΔΔ*C*_T_^ method was used to analyze the data.

### Western blot detection of protein expression in cells and myocardial tissue

The treated cells and the heart tissue were fully lysed with pre-cooled RIPA lysate, the supernatant was taken and the protein concentration was determined by BCA kit, and one-fourth volume of protein loading buffer was added, heated at 100°C for 5 min. After denaturation, it was separated by SDS/PAGE. The protein was transferred using a PVDF membrane, and 5% skim milk was prepared to block the PVDF membrane for 1 h, and then DJ-1, PTEN, Akt, p-Akt, Bcl-2, BAX, c-caspase-3 were respectively diluted at a ratio of 1:1000 primary antibody for overnight incubation at 4°C. Then, the fluorescent secondary antibody IRDye800CW (LI-COR, U.S.A.) was diluted at a ratio of 1:10000, and the PVDF membrane was incubated at room temperature for 1 h, and TBST was washed three times for 10 min each time. The corresponding gray value was measured by Odyssey Infrared Imaging Technique, and the internal reference of each protein was GAPDH.

### Flow cytometry to detect apoptosis rate of each group

Each group of treated H9c2 cardiomyocytes were digested with trypsin, centrifuged at 300×***g*** for 10 min. The supernatant was discarded, washed twice with PBS, centrifuged to remove supernatant. A total of 100 μl of 1× Annexin-binding buffer was added. The cells were suspended and added with Annexin V (5 μl) and propidium iodide (PI) 5 μl at room temperature for 20 min in the dark. Then 400 μl Annexin-binding buffer was added and mixed, stored in an ice box and detected by flow cytometry.

### DJ-1 protein and ROS fluorescence detection

The cells were given the corresponding intervention, and then washed with PBS three times for 3 min each time, fixed with 4% paraformaldehyde for 30 min at room temperature, and washed three times with PBS for 3 min each time. Goat serum was added dropwise to the slide, blocked at room temperature for 30 min, and the primary antibody of DJ-1 (1:200) was added to the blocking solution by suction paper and incubated overnight at 4°C. The excess primary antibody on the slide was washed, and the fluorescent secondary antibody with cy3 label was incubated for 1 h at room temperature, incubated with DAPI in the dark for 5 min, the cover was washed, and the image was observed with a fluorescence microscope. The ROS fluorescence assay was performed on H9c2 cells in a logarithmic growth phase with good growth conditions, seeded on 20 mm-slides at 2 ×10^5^ per well, and cultured overnight at 37°C in a 5% CO_2_ incubator. The cells were treated according to different groups, and the corresponding treatments were given respectively. The cells were incubated at 37°C for 30 min in the dark. After the incubation, the cells were washed with fresh medium; the cells were fixed and stored, and microscopic photographs were taken. Three sheets were selected for each slice. Photographs were taken for optical density analysis of fluorescent photographs using IPP 6.0 software.

### Triphenyltetrazolium chloride determination of myocardial infarction area

At the end of reperfusion, six rats were randomly selected from each group, LAD was ligated again, and 3% Evans Blue 1 ml was injected into the left ventricular cavity. The residual blood was washed with pre-cooled saline, and the dye was distributed in the myocardium through the coronary artery. The heart was quickly removed and stored at −20°C for 2 h. The sections were cut perpendicular to the long axis of the myocardium, and the thickness was 2 mm. The cells were incubated in 1% triphenyltetrazolium chloride (TTC) buffer at 37°C for 15 min in the dark, and then placed in 4% paraformaldehyde for 30 min. The blue staining part of Evans Blue is a viable myocardial tissue called normal myocardial tissue (non-ischemic myocardium), the TTC staining area is brick red (ischemic myocardium), and the non-TTC staining area is gray (infarcted myocardium). Each layer of the slice was photographed on both sides, and the photos were analyzed by ImagePro Plus 6.0 software. The area of myocardial ischemic area and percentage of infarct area were measured as representative of the percentage of ischemic myocardium area to the left ventricular area (AAR/LV%) and the percentage of infarcted myocardium area to the area of ischemic myocardium (IA/AAR%).

### HE staining, Tunel staining and immunohistochemical detection of myocardial tissue

After reperfusion for 2 h, the apical tissue was excised, fixed in 4% paraformaldehyde solutionand sliced by paraffin embedding. Myocardial pathological morphology was observed under microscope by HE staining; myocardial cell apoptosis rate was detected by Tunel staining. DJ-1 protein expression and distribution in myocardial tissue of each group were detected by immunohistochemistry.

### Statistical analysis

Statistical analysis was performed using SPSS 17.0 software. The measurement data were expressed as means ± standard deviation. After the homogeneity test for variance, the differences between two groups were analyzed using a Student’s *t* test, and the differences between groups were evaluated using the one-way analysis of variance (ANOVA). The post hoc test was performed using the LSD test. *P*<0.05 was considered statistically significant.

## Results

### Effect of NAC on DJ-1 molecule during HG-H/R cell injury

**Figure 1:**
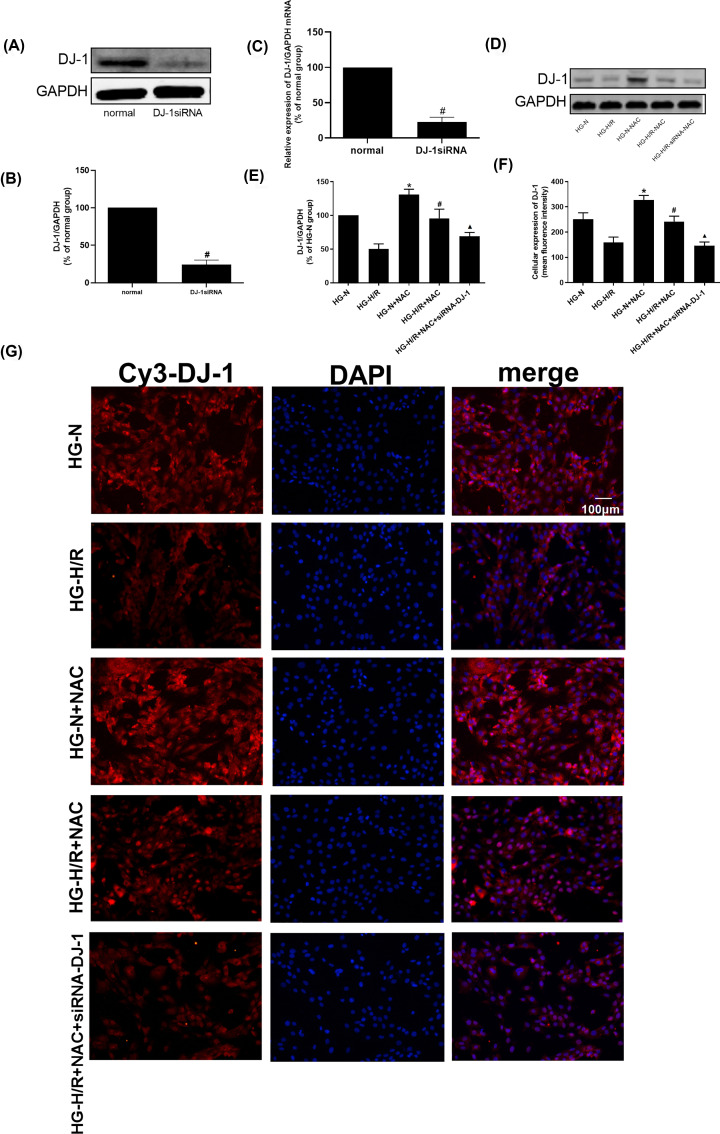
Effect of NAC on DJ-1 molecule during HG-H/R cell injury (**A**–**C**) Effect of NAC on DJ-1 protein and mRNA levels during HG-I/R cell injury. (**D,E**) The DJ-1 protein level in different groups was detected by Western blot. Results are presented by means ± S.D. *n*=3 per group. (**F,G**) Immunofluorescent analysis of DJ-1 expression in each group of cells. The cells are viewed in the field of view of 200×. (red: DJ-1; blue: DAPI). *P<0.05 compared with the HG-N group, ^#^*P*<0.05 compared with the HG-H/R or Normal group, ^▲^*P*<0.05 compared with the HG-H/R-NAC group.

SiRNA was first used to down-regulate DJ-1 in H9c2 cells. The DJ-1 protein level was detected to verify the transfection effects (Figure 1A–C), compared with normal group, the DJ-1 protein and mRNA levels were decreased in DJ-1siRNA group (*P*<0.05). As shown in Figure 1D–G, compared with HG-N group, the expression of DJ-1 protein was decreased in HG-H/R group (*P*<0.05). Compared with HG-N group, the DJ-1 protein level in HG-N+NAC group was increased (*P*<0.05). When compared with HG-H/R group, DJ-1 protein level was increased in HG-H/R+NAC group (*P*<0.05). Compared with HG-H/R+NAC group, the DJ-1 protein level in HG-H/R+NAC+siRNA-DJ-1 group was decreased (*P*<0.05).

### NAC can increase the activity of HG-H/R cell damage through up-regulating DJ-1

**Figure 2:**
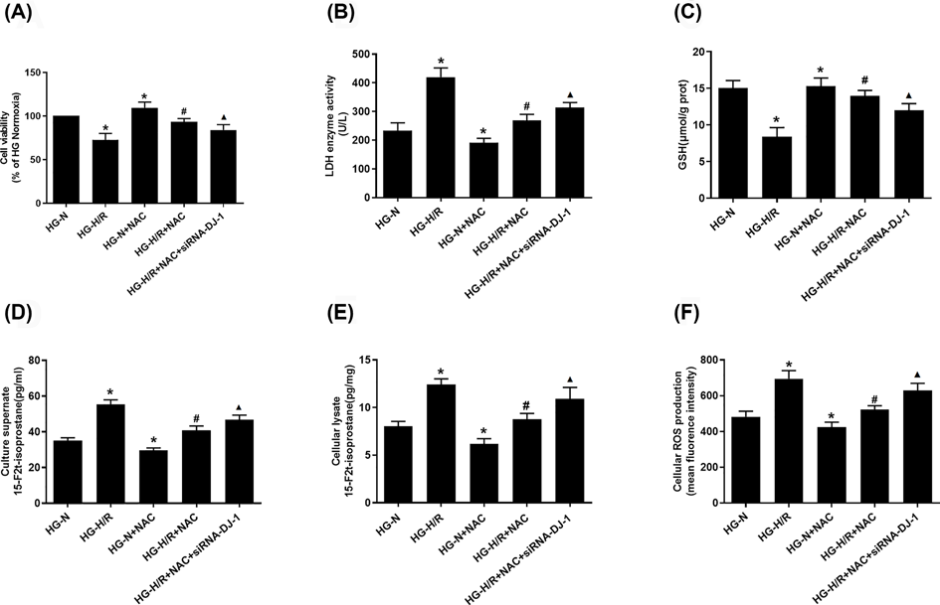
NAC increased cell activity and decreased attenuated oxidative stress damage in HG-I/R cells by DJ-1 (**A**) CCK-8 analyzes the cell viability in H9c2 cells treated with different methods. (**B**) LDH enzyme activity analyzes the degree of injury in H9c2 cells with different methods. (**C**) GSH levels were detected in different H9c2 cell groups. (**D,E**) 15-F2t-isop in cell culture supernatant and cellular levels were detected in different H9c2 cell groups. (**F**) ROS levels were revealed in different H9c2 cell groups. Results are presented by means ± S.D., *n*=6 per group. *P<0.05 compared with the HG-N group, ^#^*P*<0.05 compared with the HG-H/R group, ^▲^*P*<0.05 compared with the HG-H/R-NAC group.

As shown in Figure 2A,B, compared with HG-N group, the cell viability was decreased and the LDH level was increased in HG-H/R group (*P*<0.05). Compared with HG-N group, the cell viability increased and the LDH level decreased in HG-N+NAC group (*P*<0.05). Compared with HG-H/R group, the cell viability was increased and LDH level was decreased in HG-H/R+NAC group (*P*<0.05). Compared with HG-H/R+NAC group, the cell viability was decreased and LDH level was increased in the HG-H/R+NAC+siRNA-DJ-1 group (*P*<0.05).

### NAC attenuates oxidative stress damage in HG-H/R cells by up-regulating DJ-1

GSH is a special substance that relieves toxins. It is a small molecular peptide composed of three amino acids and acts as an important antioxidant and free radical scavenger in the body [17]. 15-F2t is the most abundant prostaglandin analog of F2-Isoprostane. This kind of substance is produced by the oxidation of unsaturated fatty acids on the cell membrane by the non-cyclooxygenase pathway. It is a specific and unique indicator of the degree of lipid peroxidation in the body [18]. As shown in Figure 2C–F, compared with HG-N group, GSH level was decreased, ROS and 15-F2t-isop levels were increased in HG-H/R group (*P*<0.05). Compared with HG-N group, GSH level was increased, ROS and 15-F2t-isop levels were decreased in HG-N+NAC group (*P*<0.05). Compared with HG-H/R group, GSH level was increased, ROS and 15-F2t-isop were decreased in HG-H/R+NAC group (*P*<0.05). Compared with HG-H/R+NAC group, GSH level was decreased, ROS and 15-F2t-isop levels were increased in HG-H/R+NAC+siRNA-DJ-1 group (*P*<0.05).

### NAC attenuates the apoptosis of HG-H/R cells by up-regulating DJ-1

**Figure 3:**
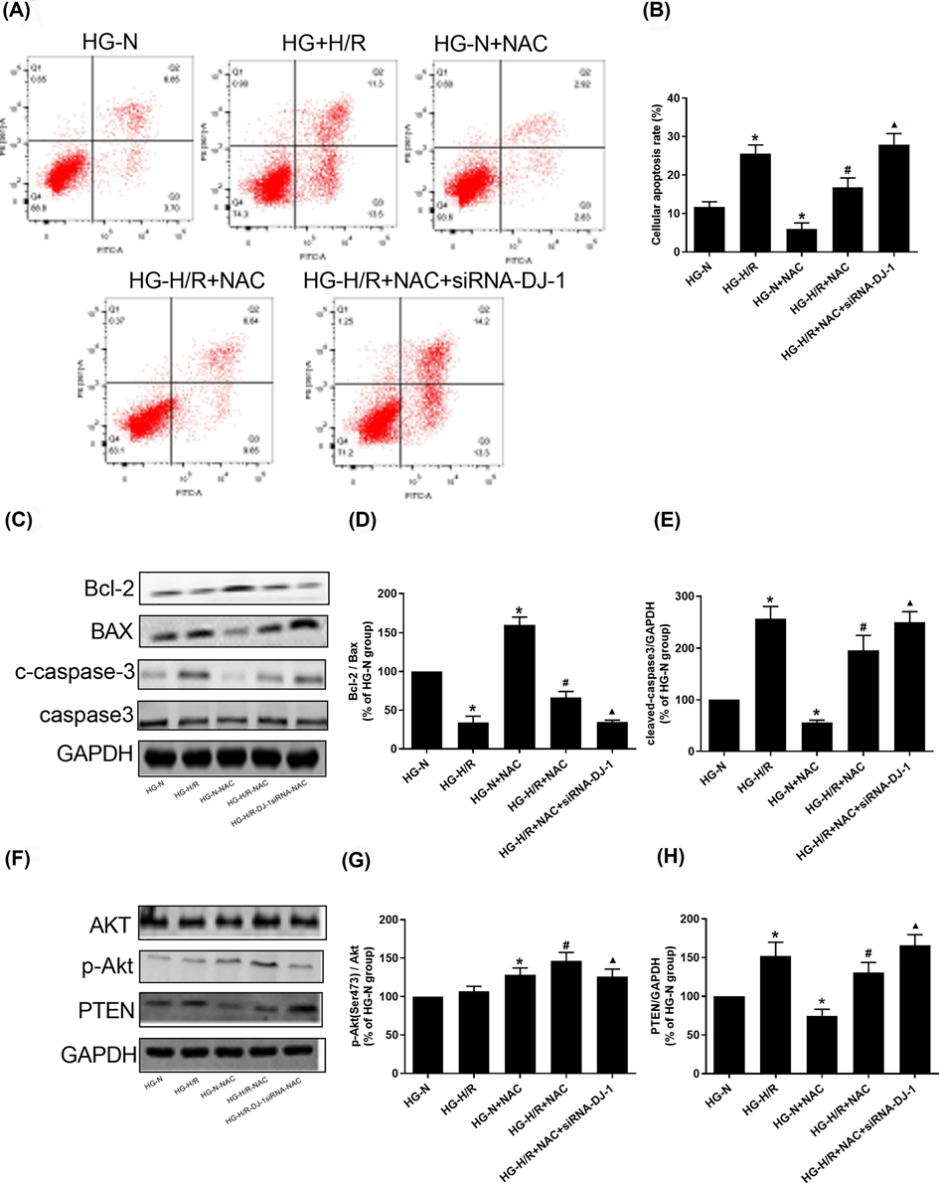
NAC attenuates the apoptosis of HG-H/R cells by DJ-1 through PTEN/Akt pathway (**A,B**) Cell apoptosis rates were detected in different H9c2 cell groups. (**C**–**E**) Bcl-2, BAX, c-caspase-3 and caspase3 protein levels were detected by Western blot in different H9c2 cell groups. (**F**–**H**) PTEN, p-Akt and Akt protein levels were detected by Western blot in different H9c2 cell groups. Results are presented by means ± S.D., *n*=3 per group. *P<0.05 compared with the HG-N group, ^#^*P*<0.05 compared with the HG-H/R group, ^▲^*P*<0.05 compared with the HG-H/R-NAC group.

As shown in Figure 3A–E, compared with HG-N group, the Bcl-2 protein level in HG-H/R group were decreased (*P*<0.05). BAX, c-caspase-3 and cell apoptosis rate levels were increased (*P*<0.05). Compared with HG-N group, Bcl-2 protein level was increased in HG-N+NAC group (*P*<0.05). BAX, c-caspase-3 and cell apoptosis rate levels were decreased (*P*<0.05). Compared with HG-H/R group, the level of Bcl-2 protein in HG -H/R+NAC group was increased (*P*<0.05). BAX, c-caspase-3 and cell apoptosis rate levels were decreased (*P*<0.05). Compared with HG-H/R+NAC group, the level of Bcl-2 protein in HG-H/R+NAC+siRNA-DJ-1 group was decreased (*P*<0.05). BAX, c-caspase-3 and cell apoptosis rate levels were elevated (*P*<0.05).

### Effect of NAC on PTEN/Akt pathway during HG-H/R-induced cell injury by regulating DJ-1

As shown in Figure 3F–H, compared with HG-N group, the level of PTEN protein in HG-H/R group was increased (*P*<0.05) and the level of p-Akt protein was slightly up-regulated in HG-H/R group, but the difference was not statistically significant (*P*>0.05). PTEN protein level was decreased in HG-N+NAC and HG-H/R+NAC group respectively compared with HG-N and HG-H/R (*P*<0.05) and p-Akt protein level was increased in HG-N+NAC and HG-H/R+NAC group respectively compared with HG-N and HG-H/R (*P*<0.05). Compared with HG-H/R+NAC, the level of PTEN protein in HG-H/R+NAC+siRNA-DJ-1 group was increased (*P*<0.05) and the level of p-Akt protein in HG-H/R+NAC+siRNA-DJ-1 group was decreased (*P*<0.05). There was no significant difference of Akt molecule in each group. There was no significant difference in the expression of caspase3 between the groups.

### Protective effect of NAC on myocardial tissue damage in diabetic I/R rats by up-regulating DJ-1

**Figure 4:**
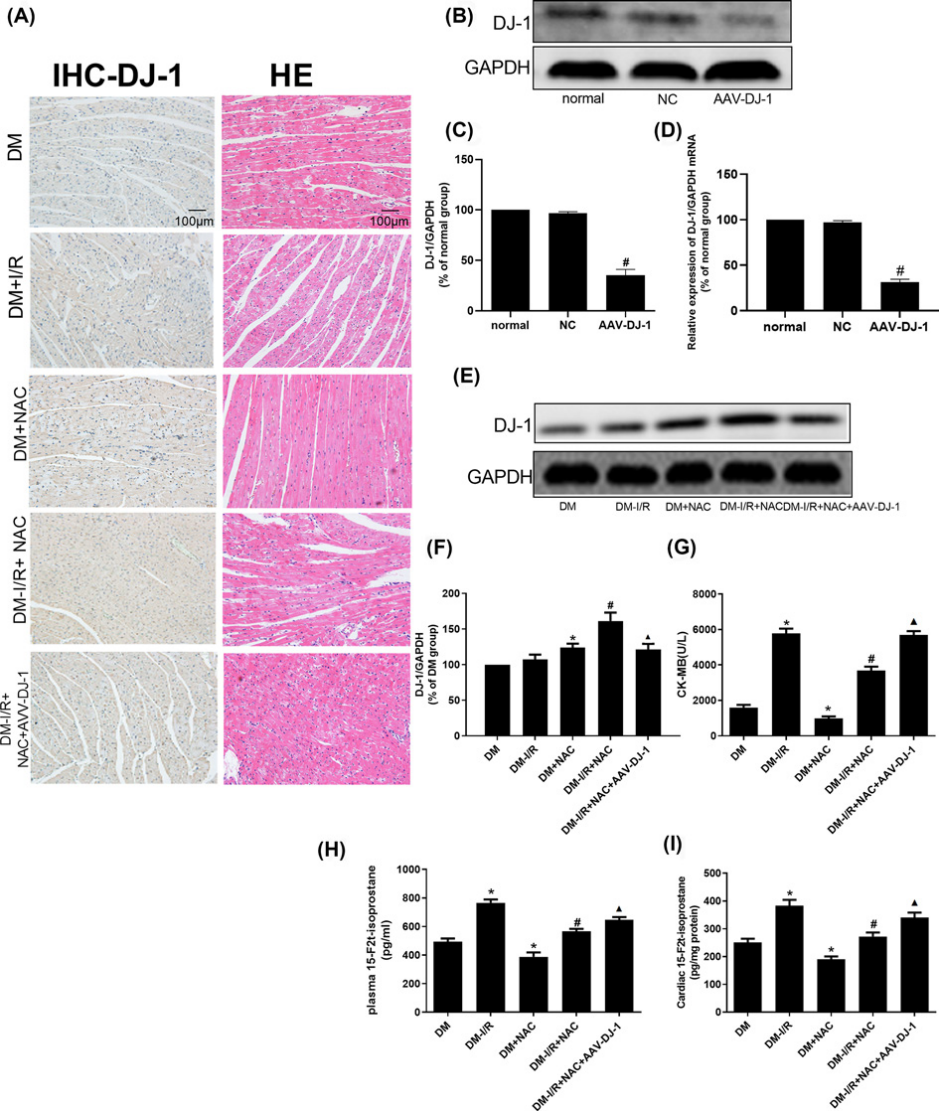
Protective effect of NAC on myocardial tissue damage in diabetic I/R rats (**A**) The pathological changes and expression of DJ-1 protein were respectively detected by HE and immunohistochemistry staining. (**B**–**D**) DJ-1 protein and mRNA levels were detected for the efficacy of AAV-DJ-1 transfection by Western blot. (**E,F**) DJ-1 protein level was detected in different rat groups by Western blot. (**G**) CK-MB levels were detected in different rat groups. (**H,I**) 15-F2t-isop for plasma and cardiac levels were detected in different rat groups. Results are presented by means ± S.D., *n*=3 per group. *P<0.05 compared with the HG-N group, ^#^*P*<0.05 compared with the HG-H/R group, ^▲^*P*<0.05 compared with the HG-H/R-NAC group.

The above experimental results verified that NAC can reduce H/R injury in HG-H/R-induced cardiomyocytes by regulating the PTEN/Akt pathway through up-regulating the level of DJ-1. Similarly, to further verify the efficacy of NAC in diabetic I/R rats, we first used the AAV to target knockdown of DJ-1 molecules in myocardial tissue, and then modeled and intervened. The general situation of each group of rats is shown in Table 1, diabetic symptoms were observed in the model group, and the heart weight to body weight, which was an indirect indicator of cardiac hypertrophy performed significant change. The efficacy of AAV-DJ-1 was determined in Figure 4B–D. Compared with normal group, the DJ-1 protein and mRNA levels in AAV-DJ-1 group were decreased (*P*<0.05). As shown in Figure 4A (for immunohistochemistry) and Figure 4E,F (for Western blot), suggest that compared with DM group, the expression of DJ-1 protein in DM-I/R group was slightly up-regulated but the difference was not statistically significant (*P*>0.05). Compared with DM group, the level of DJ-1 protein in DM+NAC group was increased (*P*<0.05). Compared with DM-I/R group, the expression of DJ-1 protein was increased in DM-I/R+NAC group (*P*<0.05). However, the expression of DJ-1 protein in the DM-I/R+NAC+AAV-DJ-1 group was lower than that in the DM-I/R+NAC group (*P*<0.05).

**Table 1:** Comparison of general conditions of rats in each group

Groups	Food intake g/(kg.d)	Water intake ml/(kg.d)	Body weight (g)	Heart weight index (%)	Blood glucose (mmol/l)
N	63.3 ± 5.6	103.8 ± 11.7	471.2 ± 17.7	3.9 ± 0.3	6.7 ± 0.8
DM	118.5 ± 7.5*	278.6 ± 15.6*	285.8 ± 20.3*	5.5 ± 0.6*	25.9 ± 4.8*

Results are expressed as mean ± standard deviation, *n*=8 compared with N (normal). *P<0.01.

As shown in Figure 4A, the results of HE staining showed that the nucleus of myocardial tissue in DM group was not uniform, some cells were swollen, a few cells were hypertrophied, the interstitial structure was not clear, the horizontal stripes were not clear, and a small amount of tissue fibers were arranged neatly. The myocardial nuclei of DM-I/R group were isolated, uneven size, hypertrophy of cardiomyocytes, unclear borders, disordered and distorted myocardial fibers, and local infiltration of inflammatory cells. The myocardial tissue of the DM+NAC group was significantly improved compared with the DM group. Myocardial tissue in the DM-I/R+NAC group was significantly better than the DM-I/R group. Myocardial tissue in the DM-I/R+NAC+AAV-DJ-1 group was significantly aggravated compared with the DM-I/R+NAC group.

### NAC attenuates oxidative stress damage in diabetic I/R rats by up-regulating DJ-1

As shown in Figure 4G–I, compared with DM group, CK-MB and 15-F2t-isop for plasma and cardiac levels in DM-I/R group were increased (*P*<0.05). Compared with DM group, CK-MB and 15-F2t-isop for plasma and cardiac levels in DM+NAC group were decreased (*P*<0.05). Compared with DM-I/R group, CK-MB and 15-F2t-isop for plasma and cardiac levels in DM-I/R+NAC group were decreased (*P*<0.05). Compared with DM-I/R+NAC group, CK-MB and 15-F2t-isop for plasma and cardiac levels in DM-I/R+NAC+AAV-DJ-1 group were increased (*P*<0.05).

### NAC attenuates the apoptosis of DM-I/R rat model by up-regulating DJ-1

**Figure 5:**
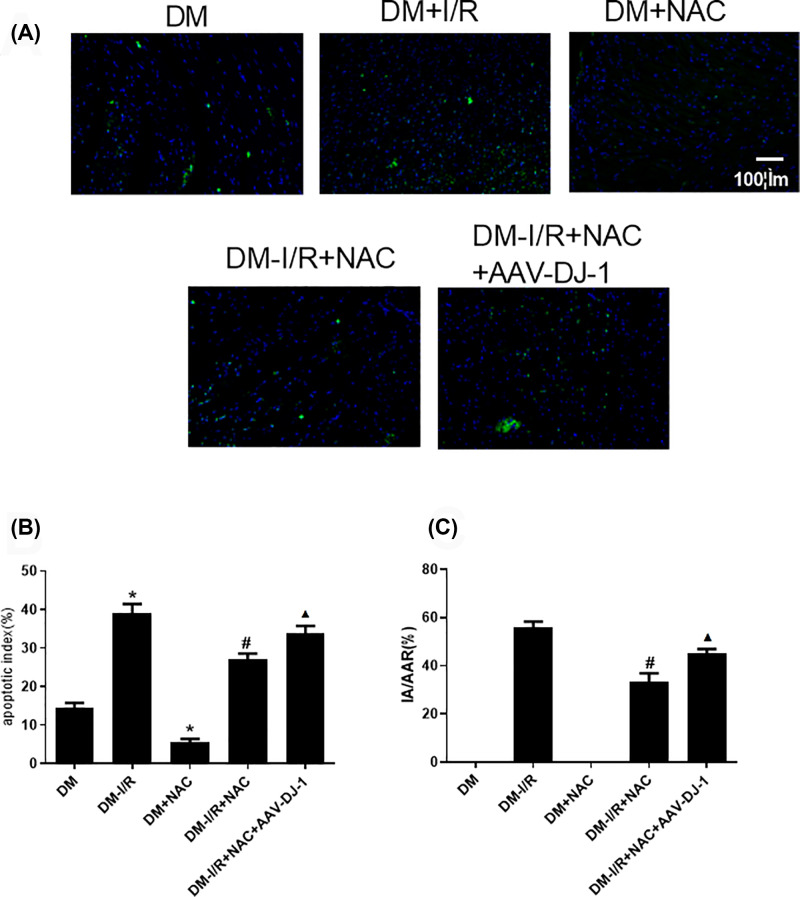
NAC attenuates the apoptosis rats and myocardial infarction area in DM-I/R rat model by DJ-1 (**A,B**) The Tunel staining for cell apoptosis rates were detected in different rat groups. (**C**) The myocardial infarction area was detected by TTC in different rat groups. Results are presented by means ± S.D. *n*=3 per group. *P<0.05 compared with the HG-N group, ^#^*P*<0.05 compared with the HG-H/R group, ^▲^*P*<0.05 compared with the HG-H/R-NAC group.

As shown in Figure 5A,B for Tunel staining on myocardial tissue, the cell apoptosis rate in DM-I/R group was increased when compared with DM group (*P*<0.05). Compared with DM group, the cell apoptosis rate in DM+NAC were decreased (*P*<0.05). Compared with DM-I/R group, the cell apoptosis rate in DM-I/R+NAC group was decreased (*P*<0.05). Compared with DM-I/R+NAC, the cell apoptosis rate DM-I/R+NAC+AAV-DJ-1 group was elevated (*P*<0.05).

**Figure 6:**
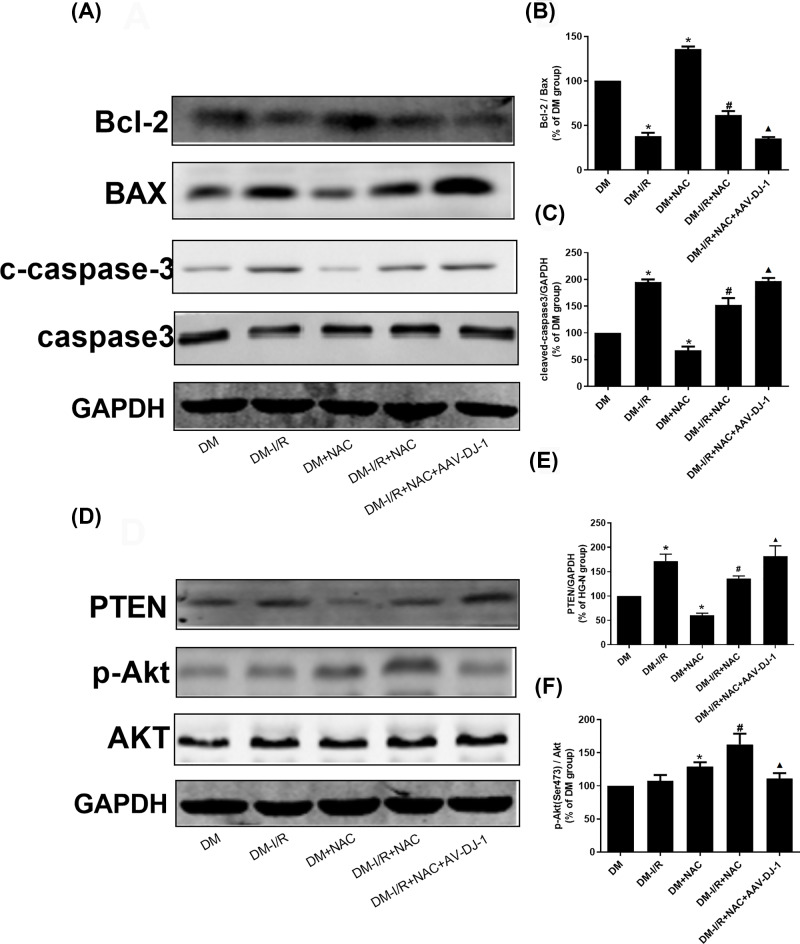
NAC attenuates the cell apoptosis in diabetic I/R rats by DJ-1 through PTEN/Akt pathway (**A**–**C**) Bcl-2, BAX, c-caspase-3 and caspase3 protein levels were detected by Western blot in different rat groups. (**D**–**F**) PTEN, p-Akt and Akt protein levels were detected by Western blot in different rat groups. Results are presented by means ± S.D. *n*=3 per group. *P<0.05 compared with the HG-N group, ^#^*P*<0.05 compared with the HG-H/R group, ^▲^*P*<0.05 compared with the HG-H/R-NAC group.

As shown in Figure 5C, Figure 6A–C and Supplementary Material, compared with DM group, the Bcl-2 protein level in DM-I/R group were decreased (*P*<0.05). BAX and c-caspase-3 were increased (*P*<0.05). Compared with DM group, Bcl-2 protein level was increased in DM+NAC group (*P*<0.05). BAX and c-caspase-3 were decreased (*P*<0.05). Compared with DM-I/R group, the level of Bcl-2 protein in DM-I/R+NAC were increased. BAX, c-caspase-3 and myocardial infarction area were decreased (*P*<0.05). Compared with DM-I/R+NAC, the level of Bcl-2 protein in DM-I/R+NAC+AAV-DJ-1 group was decreased (*P*<0.05). BAX, c-caspase-3 and myocardial infarction area were elevated (*P*<0.05). There was no significant difference in the expression of caspase 3 between the groups.

### NAC inhibits the PTEN/Akt pathway through promoting DJ-1 in DM-I/R rat model

As shown in Figure 6D–F, compared with DM group, the protein level of PTEN in DM-I/R group was increased (*P*<0.05), DJ-1 and p-Akt protein levels were slightly up-regulated but the difference was not statistically significant (*P*>0.05). Compared with DM group, PTEN protein level in DM+NAC group was decreased (*P*<0.05), p-Akt protein level was increased (*P*<0.05). Compared with DM-I/R group, PTEN protein level was decreased in DM-I/R+NAC group (*P*<0.05), p-Akt protein level was increased (*P*<0.05). Compared with DM-I/R+NAC group, the protein level of PTEN in DM-I/R+NAC+AAV-DJ-1 group was increased (*P*<0.05), p-Akt protein level was decreased (*P*<0.05). There was no significant difference for Akt molecule in each group.

## Discussion

The 2015 International Diabetes Conference focused on the high incidence of diabetes and its prevention and treatment of cardiovascular complications, two important critical medical conferences. One of the common concerns is the prevention and molecular mechanism of perioperative myocardial I/R injury. Recent epidemiological investigations still show that ischemic heart disease is the main cardiovascular complication of diabetes patients and the leading cause of death [19], again indicating the further study of the urgency of IR injury mechanism and prevention and treatment of diabetic myocardium. Evidence-based medicine studies have shown that the incidence of myocardial ischemia in diabetic patients is significantly higher than non-diabetic patients, and I/R vulnerability increases, the prognosis is worse and mortality is higher [20]. A large number of studies suggest that the I/R vulnerability of diabetic myocardium is associated with a variety of mechanisms that cause damage [21–23]. However, clinical trials in recent years have shown that the efficacy of targeted anti-oxidation and anti-inflammatory measures needs to be improved and it is worth rethinking. In view of the related research on the relationship between myocardial I/R vulnerability and main endogenous protective mechanisms, it is necessary to further clarify the changes of endogenous protective mechanisms and main regulatory mechanisms in the pathological process of diabetic myocardial I/R injury, from enhancing endogenous protective mechanism also inhibits the mechanism of injury mechanism, finds more effective prevention measures, and improves the prognosis. It is an important issue that needs to be solved in relevant disciplines.

Oxidative stress is a key link in myocardial I/R injury [24], which is mainly induced by ROS derived from explosive production during reperfusion. It exceeds the capture and clearance ability of the intracellular antioxidant defense system. Enzymes and cellular components in cells are oxidized, causing a series of damages such as protein denaturation, enzyme inactivation, DNA damage, apoptosis and calcium overload [25]. DJ-1 protein was first reported by Japanese scholar, Nagakubo et al. in 1997 [26], and the molecule is highly expressed in the brain, heart, kidney, prostate and other parts [27]. Further functional studies have shown that DJ-1 has important regulation in biological activities such as cell transformation [26], oxidative stress [28], regulation of apoptosis [29], molecular chaperone [30] and androgen receptor regulation [31]. In recent years, the role of DJ-1 protein in cell survival and oxidative stress has been highly concerned. Inden et al. found that DJ-1 overexpression significantly increased the tolerance of neuronal cells to oxidative stress damage [32]. Yanagisawa et al. found that pre-administration of exogenous GST-DJ-1 inhibits oxygen free radical production, reduces infarct size, and promotes neurological recovery during cerebral I/R injury [33,34]. Martinat et al. found that embryonic stem cells deficient in DJ-1 gene increased with the accumulation of oxidative stress, and the sensitivity of dopaminergic neurons differentiated by them to oxidative stress increased [35]. By changing the expression level of DJ-1 gene in rat dopaminergic neuron cell line and primary cultured dopamine cells, Zhou et al found that DJ-1 can protect cells against H_2_O_2_ and 6-hydroxydopamine (6-OHDA)-induced cells apoptosis [36]. Baulac et al. found that the sensitivity of the embryo to H_2_O_2_ and apoptosis were increased in the zebrafish DJ-1 knockout model [37]. Studies have shown that the oxidative stress response in the body activates the expression of DJ-1 and its downstream antioxidant stress proteins [21,38]. DJ-1 overexpression can alleviate the oxidative stress induced by I/R in H9c2 cells [39]. Jain et al. found that DJ-1 knockout mice treated with STZ had significantly lower insulin concentrations and significantly higher fasting glucose concentrations and the apoptosis rate was twice higher than that of wild-type mice [40]. Xue et al. found that in the model of myocardial hypertrophy, DJ-1 knockout mice could increase cardiac hypertrophy by regulating autophagy through mTORC1 and mTORC2 [41]. Shimizu et al. found that in the model of myocardial I/R in DJ-1 knockout mice, abnormal mitochondrial fission could occur to promote ROS production and aggravate the occurrence of myocardial cell apoptosis [42]. Billia et al. found that the production of ROS was increased in the DJ-1 knockout mice, which obstructed the transmission of respiratory chain and resulted in mitochondrial dysfunction and increased susceptibility to myocardial failure [43]. Similarly, our previous study found that DJ-1 plays a beneficial role in myocardial I/R in diabetes [21], and its specific mechanism may be generated by the AMPK/mTOR signaling pathway [44]. Therefore, DJ-1 protein may play a key role in enhancing intracellular antioxidant defense ability against oxidative stress. If DJ-1 can be used as a therapeutic target, it can effectively alleviate myocardial tissue injury during myocardial I/R.

An important pathophysiological mechanism of damage to diabetic myocardial I/R injury is the inactivation of the PI-3K/Akt pathway [45]. Siddall et al. showed that PTEN, a major negative regulator of the PI-3K/Akt signaling pathway, increased in the diabetic state, specifically dephosphorylation of the 3′-position of phosphatidylinositol 3,4,5-triphosphate. Thus blocking the PI-3K/Akt signal transduction pathway, resulting in decreased PI-3K/Akt pathway activity, thereby reducing or blocking the protective effector molecules downstream of Akt, aggravating diabetic myocardial injury [46]. As a negative regulator upstream of the PTEN/PI-3K/Akt signaling pathway, DJ-1 has anti-oxidative stress and molecular chaperone properties and plays an important role in various physiological and pathological changes *in vivo*. In the HG state, the level of DJ-1 protein is elevated, which is a negative regulator for PTEN [47]. Meanwhile, the expression of PTEN in the diabetic heart muscle is increased [48]. PTEN can furtherly inhibit the activity of the PI-3K/Akt signaling pathway [48]. For further study of myocardial I/R injury, the mechanism of damage provides a suitable entry point.

NAC is a product of acetylation of L-cysteine, it acts as an antioxidant and a thiol-based donor to increase the antioxidant enzymes such as intracellular reduced GSH. The mechanism of action of NAC may be that NAC contains a sulfhydryl group, which induces aggregation of intracellular GSH by deacetylation, promotes antioxidation, enhances intracellular detoxification and reduces release of oxygen free radicals, thereby blocking the second messenger effect of ROS. Unlike other antioxidants, NAC regulates the activity of activator protein-1 (AP-1) and the secretion of cytokines, exerting antioxidant and immunomodulatory activities. At the same time, NAC can also enter into white blood cells to convert into physiological antioxidants, increase the level of reduction in cells, and inactivate reactive oxygen in white blood cells and medium [49]. The content enhances the body’s ability to scavenge free radicals, regulates the metabolic activity of cells and prevents DNA damage, thereby exerting anti-oxidative stress [50]. The study found that NAC can reduce cardiac I/R injury through anti-oxidation and anti-apoptosis [15]. As described in our previous study, the eNOS/nitric oxide (NO) and ROS balance is important in the progression of diabetic cardiomyopathy and myocardial I/R injury in diabetes [51,52]. The data suggested that inhibition of excessive oxidative stress by NAC could restore Cav-3 expression and improve eNOS/NO signaling, which ultimately attenuate diabetic cardiomyopathy and myocardial I/R injury in diabetic rats [18]. In our study, when genetic mutation occurred or DJ-1 protein level decreased, cellular antioxidant capacity was reduced. Thus, the sensitivity of cells to oxidative stress was increased. The homeostasis of intracellular redox was out of balance and great quantities of ROS accumulated, which in turn lead to oxidative stress. Therefore, based on the same function on reducing the accumulation of ROS and decreasing oxidative stress injury, the effect of NAC on DJ-1 protein was parallelly studied with caveolin-3/eNOS signaling pathway. There is no corresponding study on the effect of DJ-1 on the regulation of PTEN/Akt pathway affecting DM-I/R. Therefore, this experiment uses DM-I/R as a model, with NAC as an intervention agent, targeting DJ-1, and observing whether NAC can alleviate diabetic myocardial I/R injury by up-regulating DJ-1 to regulate PTEN/Akt pathway.

For the results, in the *in vitro* experiments, DJ-1 in rat myocardial H9c2 cells was first knocked down by siRNA. Then the cells and rats were built as HG and hypoxia reoxygenation/ischemia reperfusion models. NAC drug intervention was used to intervene the cells. *In vitro* experiments, compared with HG-N group, H/R injury can aggravate oxidative stress injury and apoptosis rate of myocardial cells, down-regulate the expression of DJ-1 molecule, and activate the PTEN/Akt pathway. However, for the groups of HG-N and HG-H/R, NAC can significantly reduce oxidative stress injury and apoptosis rate of myocytes, promote the expression of DJ-1 molecule, and inhibit PTEN/Akt pathway. Compared with the groups of HG-N+H/R+NAC and DM+I/R+NAC, the oxidative stress injury and apoptosis rate of myocardial cells tissues increased after the targeted knockdown of DJ-1. The expression of DJ-1 molecule was inhibited, and the PTEN/Akt pathway was subsequently activated. Similarity, in the *in vivo* experiments, DJ-1 in rat cardiac tissue was knocked down by siRNA adeno-associated viral vector. Then the rats were built as diabetes mellitus and I/R models. In the DM and DM+I/R groups, NAC could significantly reduce oxidative stress injury and apoptosis rate of myocytes, promote the expression of DJ-1 molecule, and inhibit PTEN/Akt pathway. Compared with DM+I/R+NAC group, the oxidative stress injury and apoptosis rate of myocardial cells in heart tissues were increased after the targeted knockdown of DJ-1. The expression of DJ-1 molecule was inhibited, and the PTEN/Akt pathway was activated. It's worth noting that *in vivo* experiments, compared with DM group the expression of DJ-1 in DM-I/R group had a slight increase (no statistical difference). The above result was different with the i*n vitro* experiments. *In vitro* experiments, compared with normal cells the level of DJ-1 increased after HG stimulation, because HG stimulation could reduce the compensatory protective stress response [53]. After H/R, the above compensatory reaction disappeared and the level of DJ-1 decreased [53]. *In vivo* experiments, the diabetic model was a long-term pathological process. Therefore, compared with normal animals, the level of DJ-1 in diabetic rats was decreased. And the level of DJ-1 protein was slightly increased after diabetic myocardial I/R, but there was no statistical significance, which was coincidence with the previous study [44]. Moreover, NAC is an N-acetyl derivative of cysteine, containing free sulfhydryl groups that reduce disulfide bonds. As a donor for GSH, it can improve the reduction ability of GSH [54]. Therefore, NAC is widely used as an antioxidant to eliminate ROS levels. Studies suggested that the increased level of DJ-1 could reduce the oxidative stress damage of cells or tissues [39,40]. In general, high levels of GSH and low levels of ROS affected by NAC could feedback restore the level of DJ-1. DJ-1 is a protective protein against cell damage, and when the expression of DJ-1 is inhibited, the cells show more characteristics of damage whether or not they are treated with NAC (or any other antioxidant). In *in vitro* and *in vivo* results, since the NAC-treated group showed less damage characteristics than the untreated group, and the DJ-1 protein showed more damage after inhibition, we concluded that the protective effect after NAC treatment may be mediated by up-regulation of DJ-1. However, as we have previously reported, NAC as an antioxidant can also protect diabetic myocardium by regulating the caveolin-3/eNOS signaling pathway [16], so the effect of NAC may or may not only depend on DJ-1.

In summary, NAC can attenuate myocardial I/R injury by up-regulating DJ-1 level regulation of PTEN/Akt pathway, which provides new treatment and clinical basis for clinical treatment of diabetic myocardial I/R injury. However, its specific regulation of DJ-1 by NAC needs further study.

## Supplementary Material

Supplementary Material Figure

## Abbreviations

AAV: adeno-associated virus
Akt/PKB: protein kinase B
c-caspase-3: cleaved caspase-3
CK-MB: creatine kinase-MB
ECG: electrocardiograph
eNOS: endothelial nitric oxide synthase
GSH: glutathione
HE: hematoxylin-eosin
HG: high glucose
H/R: hypoxia/reoxygenation
I/R: ischemia/reperfusion
LAD: left anterior descending coronary artery
LDH: lactate dehydrogenase
NAC: N-acetylcysteine
NO: nitric oxide
PI-3K: phosphoinositide 3-kinase
PTEN: phosphatase and tensin homolog deleted on chromosome 10
SPF: specific pathogen free
TTC: triphenyltetrazolium chloride
15-F2t-isop: 15-F2t-Isoprostane
