# Supplementary material for: Mechanism of N-acetylcysteine in alleviating diabetic myocardial ischemia reperfusion injury by regulating PTEN/Akt pathway through promoting DJ-1
Source: Biosci Rep. 2020 Jun 4;40(6):BSR20192118. doi: 10.1042/BSR20192118 (PMC7273917; doi:10.1042/BSR20192118)
Supplement: Supplementary Material Figure [file BSR-2019-2118_supp.pdf]

## Supplemental material

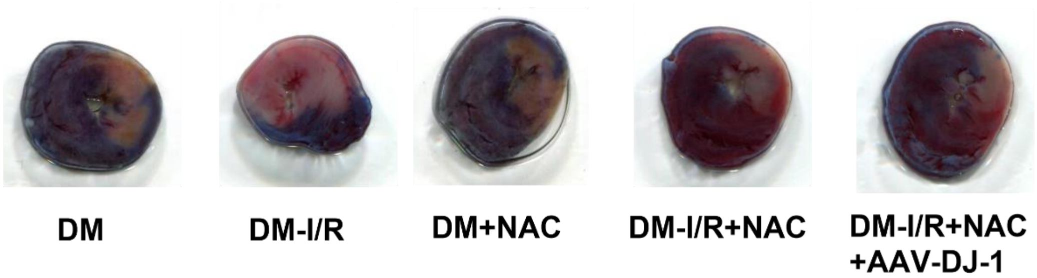


NAC attenuates the myocardial infarction area in DM-I/R rat model by DJ-1. The myocardial infarction area was detected by Evens blue-TTC double staining in different rat groups. $^{\#}P < 0.05$ compared to the DM-I/R group, $^{\blacktriangle}P < 0.05$ compared to the DM-I/R+NAC group.
